# Pomegranate Peels and Seeds as a Source of Phenolic Compounds: Effect of Cultivar, By-Product, and Extraction Solvent

**DOI:** 10.1155/2022/9189575

**Published:** 2022-07-18

**Authors:** Lara Campos, Luana Seixas, Marta H. F. Henriques, António M. Peres, Ana C. A. Veloso

**Affiliations:** ^1^Polytechnic Institute of Coimbra, Coimbra Agriculture School, Bencanta, 3045-601 Coimbra, Portugal; ^2^CERNAS-Research Centre for Natural Resources, Environment, and Society, Coimbra Agriculture School, Bencanta, 3045-601 Coimbra, Portugal; ^3^CEB-Centre of Biological Engineering, University of Minho, Campus de Gualtar, 4715-057 Braga, Portugal; ^4^Polytechnic Institute of Coimbra, Coimbra Institute of Engineering, Rua Pedro Nunes-Quinta da Nora, 3030-199 Coimbra, Portugal; ^5^Centro de Investigação de Montanha (CIMO), ESA, Instituto Politécnico de Bragança, Campus de Santa Apolónia, 5300-253 Bragança, Portugal; ^6^LABBELS-Associate Laboratory, Braga/Guimarães, Portugal

## Abstract

The nutraceutical properties of *Punica granatum* L. are not restricted to the edible portion of the fruit but also to the peels and seeds, flowers, leaves, and tree bark. The recovery and valorization of the peel and seeds (ca. 50% of the whole fruit), besides the positive environmental impact, can be viewed as a source of natural bioactive compounds. Thus, the bioactive properties of extracts of pomegranate peel and seeds from Acco and Wonderful known cultivars, as well as of the novel Big Full cultivar, were evaluated. The dried and ground pomegranate by-products were submitted to a conventional solid/liquid extraction with ethanol/water mixtures (0%, 25%, 50%, and 75% of EtOH, v/v). The obtained extracts were characterized in terms of total phenolic compounds (TPC), total flavonoids (TF), and antioxidant activity (AA), determined by the DPPH radical scavenging activity and expressed as IC_50_ (half maximum inhibitory concentration). With the exception of the Acco cultivar, the extraction yield (EY) was higher for peels, whose extracts showed higher TPC, TF, and IC_50_ (lower AA). The extracts obtained from the by-products of the Big Full cultivar had a statistically higher overall bioactive potential (TPC: 0.36 mg GAE/mg extract; TF: 0.031 mg CATE/mg extract; IC_50_: 0.51 mg/mL) compared to the other two studied cultivars. Furthermore, the EY was enhanced by solvents richer in ethanol (50-75%), allowing obtaining extracts richer in TPC and TF with higher AA. Finally, it was shown that EY combined with bioactive data allowed a satisfactory principal component unsupervised differentiation of the pomegranate extracts according to the type of by-product used.

## 1. Introduction

Pomegranate (*Punica granatum* L.) belongs to the class Magnoliopsida, order Myrtales [1, 2]. With more than 500 cultivars distributed worldwide [3, 4], this plant is considered native from Central Asia [5] and one of the most important crops in tropical and subtropical areas, due to the low maintenance cost, good productivity yields, and the ability to thrive with limited humidity [1, 4, 6]. In Portugal, the pomegranate production area has risen in the last decade reaching 475 ha, in 2018, with a total production of 3 Mtons and a productivity of 6000 kg/ha [7].

Traditional medicine recognizes pomegranate as a source of natural antiviral, antifungal, antibacterial, anthelmintic, anti-inflammatory, and antioxidant compounds [8, 9]. Pomegranate's antioxidant activity depends on several factors, like cultivar, part of the plant (fruit, flower and leaf), part of the fruit (arils, seeds and peels), climatic conditions, and ripening stage [6]. Pomegranate is mainly consumed fresh or in the form of fruit juices, jellies, and jams [10]. The entire fruit is considered a significant source of dietary phytochemicals, and their extracts can also be used as dietary supplements or ingredients in medical applications [4, 6, 9, 11–14]. Of the whole fruit, on average, the amount of pomegranate juice varies between 38 and 50%, the peels represent 39-53%, and the seeds represent 8-12% [2, 4]. Thus, it is not surprising that pomegranate processing generates large amounts of solid by-products that are generally viewed as nonvaluable waste [10, 15, 16].

Mphahlele et al. [17] clearly highlighted the potentialities of using pomegranate peels and seeds as sources of natural bioactive compounds and functional ingredients, which could be further applied in the food, pharmaceutical, and other fields. These bioactive compounds include phenolics (flavonoids, anthocyanins, and ellagitannins), vitamins, minerals, sterols, dietary fibers, and fatty acids [2, 18, 19] and have been proposed as nutraceuticals and preservatives, replacing synthetic food additives [20]. It has also been shown that pomegranate phenolic compounds are primarily responsible for its bioactivity [6, 9, 18, 19, 21]. Despite this, most of the pomegranate by-products is still discarded as waste [10, 22], without undergone any recycling or valorization process, presenting serious environmental problems [16].

Recent studies pointed out that pomegranate peel extracts had significant antioxidant, antimicrobial, and antifungal activities, as well as in vitro cytotoxic properties, which have been attributed to the synergistic effects of their phenolic phytochemicals [10, 16, 23, 24]. Alexandre et al. [10] demonstrated the antimicrobial activity of pomegranate peel extracts against various pathogenic or contaminant microorganisms but not against lactic acid bacteria (LAB), which is advantageous since LAB are probably favorable for human health. It was also reported that, using an integrated approach, oils enriched with antioxidants could be produced using carotenoids extracted from pomegranate peels [25]. A tannin-rich dried extract from pomegranate peels was recently presented as a novel oenotannin to be used as coadjuvant in the winemaking process. The tannin extract has been shown to aid in the stabilization of white wine allowing reducing the use of sulfites during vinification [26]. Although few studies have been conducted with pomegranate seeds, some authors have shown that they contain lower levels of flavonoids, as well as lower antioxidant activity than pomegranate peels [4, 9, 21, 27].

In this context, the study undertaken is aimed at investigating the potential of extracting bioactive compounds from two pomegranate by-products (peels and seeds) of the Acco, Big Full, and Wonderful cultivars, produced in Portugal. To the authors' best knowledge, no studies have been carried out on the cultivar Big Full, and there are no data available on the antioxidant activity, phenolic, and flavonoid contents of its by-products. It was also envisaged to evaluate the feasibility of using pomegranate seeds, a less studied by-product matrix, as a possible bioactive phenolic source. The effect of the percentage of ethanol in the hydroalcoholic extraction solvent used (ethanol/water mixtures), under the same extraction conditions, was evaluated on the extraction yields (EY) as well as on the total phenolic compounds (TPC), total flavonoids (TF), and antioxidant activity (AA) of extracts from pomegranate peel and seeds. Finally, the possibility of using the experimental data (EY, TPC, TF and AA) as biomarkers for by-product, cultivar, or extraction solvent differentiation was further investigated based on a principal component analysis (PCA).

## 2. Materials and Methods

Pomegranate peels and seeds from three different cultivars (Acco, Big Full and Wonderful) were dried and ground and their ethanol/water extracts submitted to a phytochemical screening before their bioactive composition was compared, through the assessment of TPC, TF, and AA. It was also intended to determine the percentage of ethanol that ensured the highest EY, as well as the highest overall bioactive potential.

### 2.1. Pomegranate Peel and Seed Recovery and Preparation

**Figure 1:**
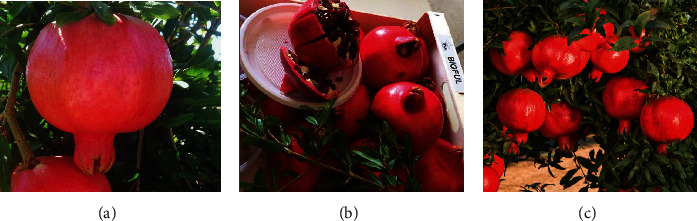
Pomegranate cultivars studied: (a) Acco, (b) Big Full, and (c) Wonderful.

POM Portugal Lda. provided pomegranate fruits from Acco, Big Full, and Wonderful cultivars (Figure 1), which were harvested in Alentejo region of Portugal (GPS coordinates 37.81717, -8.19534). All fruits were produced under the same climatic conditions and subjected to similar fertilization, irrigation, harvesting, storage, and postharvest treatments.

**Figure 2:**
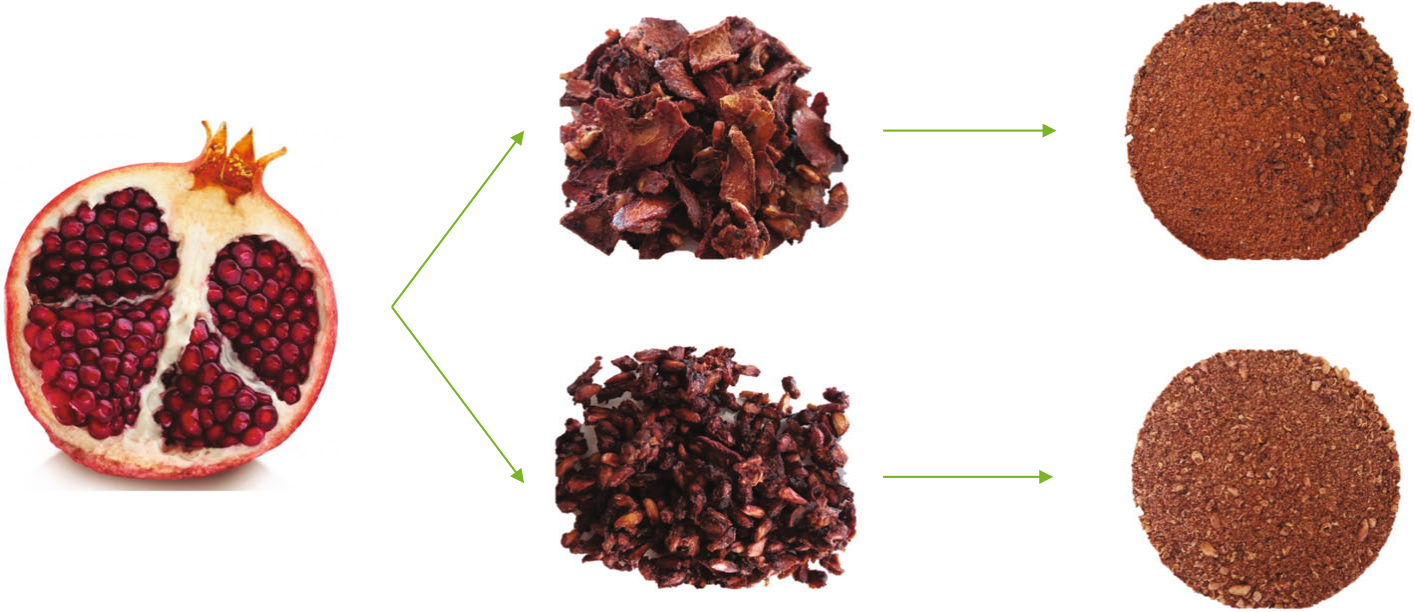
Visual aspect of the dried and ground pomegranate by-products: (a) peels and (b) seeds.

Ripe fruits were washed, sorted, and squeezed, and the obtained pomegranate by-products (peels and seeds) dried, finely ground into a powder (Figure 2), and then packed and stored at 20-25°C [28]. Before packing, the moisture and ash contents of the by-products were determined as described in the literature [29, 30].

### 2.2. Chemicals

All chemicals used for analysis were of analytical grade. For the extractions, absolute ethanol (Chem-Lab, Belgium) was used.

### 2.3. Pomegranate Peel and Seed Extraction

Four ethanol/water mixtures (0%, 25%, 50%, and 75% EtOH, v/v) were tested to evaluate the bioactive compounds' extraction capability from pomegranate peels and seeds powders. A conventional solid/liquid extraction (solid/solvent ratio of 0.02 g/mL) was conducted for 4 h, using stirred (200 rpm) sealed glass flasks, immersed in a water bath (50°C), as described by Campos et al. [28]. After extraction, the solvent was evaporated until 20 mL of solution, and the obtained extracts were freeze-dried and stored until analysis [28]. Four independent extractions were performed for each cultivar, type of by-product, and solvent. The extraction yields (EY, in %) were determined as the mass of the extract recovered from the mass of the dry matrix used for the extraction (mg of extract per 100 mg of dry by-product).

### 2.4. Qualitative Phytochemical Analysis

For qualitative phytochemical analysis, the freeze-dried extracts were resuspended in distilled water (0.02 g/mL) and screened for the presence of phytochemical families of compounds according to the methods described by Vesoul and Cock [31].

Briefly, total phenolic compounds were assessed using the Folin-Ciocalteu procedure; total flavonoids and free anthraquinones were determined by the Kumar test; tannins were evaluated through the ferric chloride test; the presence of saponins was confirmed by the detection of a persistent foam; the Ajaiyoba test was used to evaluate the presence of combined anthraquinones; alkaloids were screened using Wagner's reagent; the Leiberman-Buchard test was used to assess the presence of polysteroids; triterpenoids were evaluated using the Salkowski test; and cardiac glycosides were determined with the Keller Kiliani test.

### 2.5. Quantitative Analysis

The total phenolic compound (TPC) determination in the pomegranate by-products extracts was assessed using spectrophotometric analysis as described by Campos et al. [28] considering the Singleton and Rossi [32] method. TPC was expressed as mg of gallic acid equivalent per mg of extract (mg GAE/mg extract).

The total flavonoids (TF) were determined using spectrophotometric analysis, as described by Campos et al. [28], considering Kim et al. [33] method. TF were expressed as mg of catechin equivalent per mg of extract (mg CATE/mg extract).

The antioxidant activity (AA) of the extracts, accessed by the DPPH antiradical scavenging assay, was measured using a spectrophotometric method described by Campos et al. [28]. The results were expressed as the half-maximal inhibitory concentration (IC_50_, mg/mL).

It should be noticed that, although several mechanisms are responsible for the AA of the phenolic compounds [34], other methods are usually applied including the FRAP (ferric-reducing antioxidant), metal chelating activity, and ABTS (2,2′-azino-bis(3-ethylbenzothiazoline-6-sulfonic acid) [35]. Nevertheless, in this work, the antioxidant activity was only evaluated by the DPPH method, as recommended by Shaygannia et al. [36], who referred that pomegranate peels and seeds have a high content of punicalagin that inhibits superoxide and DPPH free radicals.

All analyses were performed in duplicates of two independent assays.

### 2.6. Statistical Analysis

A three-way ANOVA with interactions was applied to evaluate the statistical significance of the three main effects (pomegranate by-product, pomegranate cultivar, and extraction solvent-percentage of EtOH in the solution) on the EY, TPC, TF, and AA. Tukey's multiple range test and/or the estimated marginal mean plots were further used to infer about which factor's level influenced the dependent variables under study, depending on the statistical significance of the interaction effects and on the type of interaction found (additive or nonadditive/disordinal effect) [37].

Principal component analysis (PCA) was further used to evaluate the overall capability of the TPC, TF, AA, and the EY data, determined based on conventional analytical techniques, to allow an unsupervised differentiation of the extracts according to the pomegranate by-product (peels or seeds), the pomegranate cultivar (Acco, Big Full, and Wonderful), or the extraction solvent used. All statistical analyses were performed using the Sub-select [38] and MASS [39] packages of the open-source statistical program *R* (version 2.15.1), at a 5% significance level.

## 3. Results and Discussion

### 3.1. Pomegranate Peel and Seed Characterization and Phytochemical Screening

Pomegranate by-products have a high moisture level, and so their storage requires the application of a conservation method. Drying is a key step in reducing the moisture content in order to preserve the pomegranate by-products, extending their shelf life and affecting the physical and chemical changes of their added value chemicals like the bioactive compounds, which have significant commercial and industrial relevance. The drying process applied to the peels and seeds of the three studied cultivars allowed obtaining dried matrices, with a final moisture content of 10.1-19.1% for peels and 14.1-21.6% for seeds (Table 1). In general, the moisture contents of the dried peels and seeds of Acco, Big Full, and Wonderful cultivars were greater than those reported in the literature for peels and seeds from different cultivars, after being subjected to different time-temperature drying conditions. Güzel and Akpinar [40] and Ullah et al. [41] reported a moisture content of 10.3% and 4.0%, respectively, for dried peels (drying process: 48 h at 60°C and 50°C, respectively). Jalal et al. [42] reported a moisture content of 12.5% for peels and of 5.8% for seeds, dried at 60°C for 12 and 6 h, respectively. Rowayshed et al. [43] reported moistures of 13.7% for peels and 5.8% for seeds (dried at 60°C for 6 and 24 h, respectively). These authors did not specify the studied cultivars, which is known to be a key factor.

Regarding the qualitative phytochemical screening (Table 2), performed for all solvents, it was observed that all the extracts contained TPC and TF. Tannins were also present in all extracts with the exception of those from Big Full seeds with water as solvent. Saponins were detected in peels' extracts obtained with EtOH 0%, EtOH 25%, and EtOH 50%. Only Big Full seeds showed the presence of free anthraquinones; and combined anthraquinones were mostly observed in Acco and Big Full seed extracts. In general, the extracts contained triterpenoids, and only some EtOH 50% and EtOH 75% extracts tested positive for cardiac glycosides. Alkaloids and polysteroids were not identified in the studied matrices.

Elfalleh et al. [9] also performed a qualitative phytochemical screening (TF, Tan, Sap, and Alk) on pomegranate's peels and seeds of Gabsi cultivar, using water and methanol as solvents. Regardless of the solvent, the results of our study are in-line with those previously reported [9], namely, regarding a higher presence of Flav and Tan in peels than seeds. For saponins, they found that both peels and seeds are rich in these compounds, contrary to our results (Table 2), being Sap mainly detected in peels. Also, in our study, alkaloids were not detected in the extracts; however, these compounds were found in peels and seeds by the abovementioned researchers [9].

Overall, our results suggest that depending on the solvent, the phytochemicals can be extracted in different amounts. Also, differences were found between peels and seeds, especially regarding saponins and free and combined anthraquinones. The phytochemical screening performed allowed to focus the quantitative tests for assessing only the contents of total phenolic compounds and flavonoids, since these were the compounds present in all extracts.

### 3.2. Pomegranate Peel and Seed Bioactive Composition

Conventional solvent extractions were carried out using four different mixtures of ethanol/water (0%, 25%, 50%, and 75% EtOH, v/v), which were chosen since polyphenols are soluble in alcoholic solvents due their polar nature [18]. In addition, aqueous ethanol solutions were used for extractions, as they are food grade quality, with ethanol being considered a green solvent compared to other solvents (e.g., methanol). Although extraction time and temperature also influence the extraction purity and yield [44, 45], in the present work, both parameters were set to 4 h and 50°C.

The extraction yields (EY), total phenolic compounds (TFC), total flavonoids (TF), and the antioxidant activity (AA) of the peels and seeds of the analyzed pomegranate cultivars (Acco, Big Full, and Wonderful) according to the ethanol/water solvent used for extraction, are presented in Table 3. Overall, regardless the type of the solvent or cultivar, peels have higher bioactive potential than seeds (with exception for EY of Acco). Peels of the cultivar Big Full presented the highest values in terms of EY, TPC, and TF, when compared to the Acco or Wonderful cultivars, regardless of the extraction solvent (except for EY with EtOH 75%). However, Wonderful peels had the highest AA (lower IC_50_) in all ethanolic solvents tested. In addition, seeds from Wonderful appear to be better than the seeds from Acco or Big Full, independently of the extraction solvent. Extracts with less AA were obtained using water as the extraction solvent, regardless of the type of by-product.

According to the literature, the EY, TPC, TF, and AA of peels and seeds of different pomegranate cultivars, under different extraction conditions, were already evaluated. Several cultivars were studied, namely, Lefan, Katirbasi and Asinar cultivars [46]; Cekirdeksiz IV cultivar [46, 47]; Hicaznar, Cekirdeksiz VI, Fellahyemez and Ernar cultivar [47]; Dente di Cavallo cultivar [18, 48]; Natanz, Shahreza and Doorak cultivars [21]; Mollar de Elche cultivar [48]; Ganesh cultivar [49]; Pishras cultivar [50]; Settat, Beni Mellal and Berkane cultivars [51]; Mauritian cultivar [52]; as well as the Wonderful and Acco cultivars [17, 44, 48, 53, 54]. To the authors' best knowledge, no study has been carried out for Big Full cultivar. For that reason, the results reported in this work are of particular interest for their novelty to the field.

Various extraction methods have also been described in the literature, such as conventional solvent extraction [34, 47–50, 55], superheated extraction [50], high pressure extraction [10, 50, 53], supercritical extraction [23], probe-type sonication extraction [17, 25], ultrasonic-bath extraction [27, 54], and Soxhlet extraction [21], using fresh or dry samples, different solvents (water, ethanol, methanol, carbon dioxide, n-hexane, acetone, diethyl ether, ethyl acetate, sunflower oil, soya oil), extraction time (ranging from 2 min to 48 hours), temperature (from room temperature to 190°C), pressure (from atmospheric pressure to 10 MPa), particle size [56], and different solid/solvent ratios.

Comparing the extraction yields obtained in this study (25–59%) (Table 3) with those reported in literature, which used conventional extraction with water, ethanol, or their mixtures, our results are in line with the values reported by Wang et al., [44] (0.88–46.51%), Orak et al. [47] (42–61%), and Rahnemoon et al. [50] (50.10%), higher than that achieved by Masci et al. [18] (11%), Kanatt et al. [34] (13.11%), and Orak et al. [47] for the seeds (4-12%), but smaller than those reported by Sood and Gupta [57] (68%). However, it is noteworthy that the type of cultivar and the extraction conditions (time, temperature, and solid/solvent ratio), that vary in all these studies, can also significantly affect the results obtained.Overall, the TPC results for peels (0.24-0.85 mg GAE/mg) and seeds (0.08-0.23 mg GAE/mg) in this study are much higher than those reported by Sabraoui et al. [51] (peels: 0.21-0.22 mg GAE/mg; seeds: 0.06-0.07 mg GAE/mg) and Rummun et al. [52] (0.19 and 0.001 mg GAE/mg, for peels and seeds, respectively), for different cultivars and conventional extraction systems (stirring with methanol [51] and maceration with 70% methanol [52]). For TF, our results for seeds extracts (0.007-0.026 mg CATE/mg) are also higher than those reported values in the literature (0.0003 mg QE/mg [52] and 0.002 mg RE/mg [51]). Regarding the TPC in Wonderful peels, our results (0.24–0.32 mg GAE/mg) were higher than those obtained by Wang et al. [44] (8-22%) and Sumere et al. [53] (0.043 mg/mg) under high pressure conditions (10 MPa) using similar extraction solvents (0-70% of EtOH) and also than those reported by Santos et al. [54] (0.03-0.06 mg/mg) using ultrasonic bath extraction. However, Mphahlele et al. [17] that used sonication extraction with methanol reported greater contents (1.750 mg GA/mg). These authors also obtained higher levels of TF (0.095 mg/mg) compared to our results (0.030–0.043 mg CATE/mg). It should be remarked that the extraction potential of a solvent, like methanol, also depends on the extraction method applied, namely, when conventional extraction is compared to the sonication extraction, being the first less effective [30]. For the seeds of cultivars Acco and Wonderful, Falcinelli et al. [48], using n-hexane and acetone for extraction, reached TPC of 0.002 mg GAE/mg, and the TF represented ca. 99% of the TPC. These values are significantly lower than those reported in this work (0.09–0.23 mg GAE/mg for TPC). However, Falcinelli et al. [48] also found that Wonderful seeds were a better source of TPC and revealed higher AA than Acco seeds (4 *μ*mol Trolox equivalents/g against 3 *μ*mol Trolox equivalents/g), corroborating our findings.

### 3.3. Influence of Pomegranate By-Product, Cultivar, and Extraction Solvent on the Extraction Yield, Phenolics, Flavonoids, and Antioxidant Activity

Three-way ANOVA with interactions (Table 4) showed that EY, TPC, TF, and IC_50_ significantly depended on the type of pomegranate by-product (peels or seeds), on the cultivar (Acco, Big Full, or Wonderful) and on the proportion of ethanol/water used as extraction solvent (EtOH: 0%, 25%, 50%, or 75%, v/v) (*P* value <0.0001, three-way ANOVA). For all the bioactive capacity-related parameters (TPC, TF, and IC_50_), both the 2^nd^ and 3^rd^ order interaction effects were statistically significant (*P* value <0.0001, three-way ANOVA). On the contrary, for the EY only, the 2^nd^ order “by-product × cultivar” effect was statistically significant (*P* value <0.0001, three-way ANOVA). Thus, before discussing the main effects, the nature of the significant 2^nd^ order interaction effect (additive versus nonadditive/disordinal interaction) was visually checked from the estimated marginal mean plots for all the parameters. The plots for EY, TPC, and TF (not shown) revealed that in general, the 2^nd^ order interaction effects had an additive nature (no crossing lines or a slight overplotting observed), being possible to discuss each main effect separately and only based on Tukey's multiple range test (Table 4). Oppositely, nonadditive (i.e., disordinal) 2^nd^ order interaction effects were found for IC_50_ (Figure S1), imposing to discuss the main effects as a combination between the multiple range tests and the referred plots. Keeping in mind these considerations, it was observed (Table 4) that pomegranate peels had the highest EY (compared with seeds), and their extracts were significantly richer in TPC and TF than seeds' extracts (*P* value <0.0001). However, seeds had, in average, lower IC_50_ (i.e., higher AA, with the exception of Big Full cultivar or when using only water as the extraction solvent, Figure S1). The overall findings are in accordance with those reported by Orak et al. [47]; although, this latter reported significantly lower EY for seeds (varying from 4 to 12%) compared to those determined in the present study (average value of 38%). Regarding the pomegranate peels, the EY (average value of 48%) fall within the wide range of values reported in the literature (varying from 0.88 to 68%, depending on the cultivar and ethanol/water mixtures used as extraction solvent). Also, regarding peels' extracts richness in TPC and TF, the results are in agreement with the literature data for different cultivars (including Acco and Wonderful) and different extraction conditions [21, 34, 44, 47, 53, 57, 58]. It should also be remarked that a wide range of TPC (peels: 0.0018-1.75 mg GAE/mg; seeds: 0.00012-0.072 mg GAE/mg) values have been reported in the literature [21, 34, 46, 47, 57]. Thus, the results found in this study for peels (average of 0.41 mg GAE/mg) were of the same magnitude of the highest ones reported and far greater than seeds (0.15 mg GAE/mg). Regarding TF, few data is available and does not allow a straightforward comparison since the values are reported in rutin or quercetin equivalent. The higher AA of seeds' extract compared to peels' extract, found in this study, disagree with the literature [4].

Regarding the pomegranate cultivar effect (Table 4) the highest EY was obtained for Acco cultivar (51 ± 6%), followed by Big Full (41 ± 9%) and Wonderful (38 ± 11%) cultivars. On the other hand, Big Full had significantly (*P* value <0.0001) greater overall TPC, TF, and AA (i.e., lower IC_50_, which is more evident if the extraction was performed only with water, as can be inferred from Figure S1), being the opposite behavior observed when the solvent contained EtOH, followed by Wonderful and Acco cultivars, demonstrating the potential production interest on this most recent and less-studied pomegranate cultivar. The highest bioactive potential of Wonderful cultivar compared to Acco is in accordance with that reported in literature for the juices of these both cultivars [3] and was attributed to the high acidity of Wonderful pomegranate fruits [3, 59], which is responsible for higher hydrophilic and lipophilic antioxidant capacity [13]. Indeed, it has been suggested that less acidic pomegranate cultivars have lower phenolic contents, possessing lower antioxidant capacity [11], since higher contents in organic acids enhance the antioxidant potential [13].

Lastly, the results also pointed out (Table 4) that the relative proportion of ethanol on the extraction solvent influenced the extraction yield as well as the bioactive parameters. In fact, EtOH 50% was the extraction solvent that promoted, as a whole, the highest average EY (45%), TFC (0.34 mg GAE/mg), and TF (0.026 mg CATE/mg), as well as the lowest IC_50_ (0.12 mg/mL; and so, the greatest AA), regardless of the by-product or the pomegranate cultivar. It was also clear that applying aqueous extraction resulted in the lowest EY, originating extracts with the poorest bioactive capacity. These overall findings agree with those reported by Masci et al. [18], Kanatt et al. [34], and Sood and Gupta [57]. Other studies also concluded that the best ethanol/water extraction solvent had a 50 : 50 (v/v) ratio [49, 50, 56]; although, some researchers also verified that the extraction with other solvents, like for example, methanol/water mixtures, could lead to better results [44, 49, 58].

Finally, the abovementioned findings must be further evaluated for other extraction conditions (e.g., solid/solvent ratio, extraction time, temperature, solvent or extraction technique), due to their known influence on the bioactive quality of the obtained pomegranate by-products extracts [44, 50, 53–55].

### 3.4. Principal Component Analysis (PCA): Bioactive Parameters and Extraction Yield as Potential Pomegranate By-Product, Cultivar, and/or Extraction Solvent Markers

**Figure 3:**
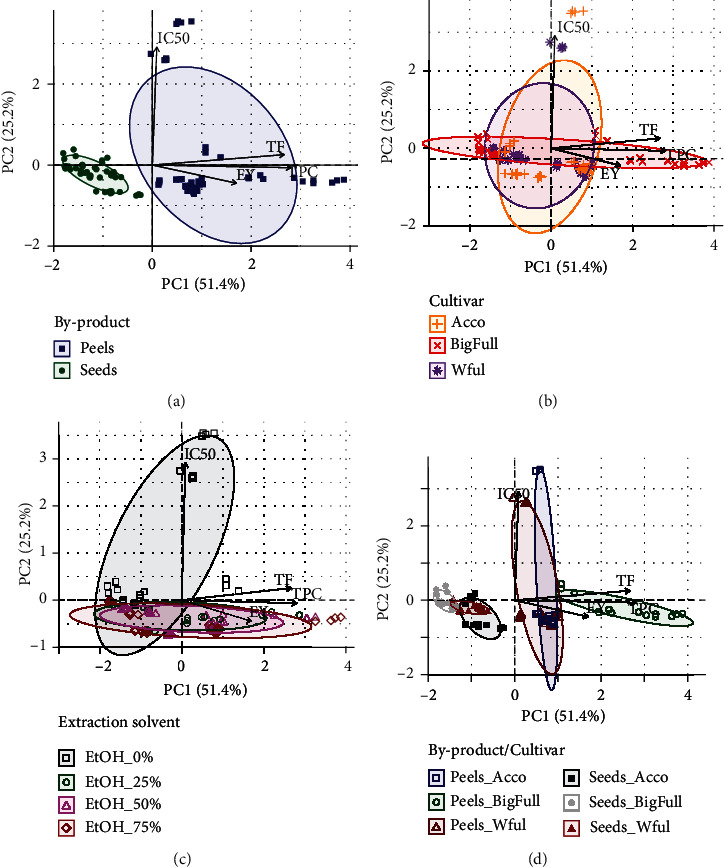
2D-PCA plots for the pomegranate extract classification based on the experimental extraction yields (EY), total phenolic compounds (TPC), total flavonoids (TF), and antioxidant activity (AA assessed in terms of IC_50_) according to the following: (a) the type of by-product (peels or seeds), (b) the cultivar (Acco, Big Full, and Wonderful), (c) the type of extraction solvent (EtOH: 0%, 25%, 50%, 75%), and (d) the by-product and the cultivar simultaneously.

As pointed out, the extraction yield and the bioactive overall potential of the extracts evaluated are highly dependent on the pomegranate cultivar, by-product matrix, and the ethanol/water mixture. The three main factors studied influenced in different ways the composition of the extracts, highlighting that both peels and seeds by-products were a potential source of bioactive compounds, namely, phenolics, like flavonoids, possessing a satisfactory antioxidant capacity. Thus, PCA was further applied aiming to verify if the EY, TPC, TF, and IC_50_ data could be successfully used to establish an unsupervised differentiation of the extracts according to each of the three main effects, individually. As can be seen from Figure 3 (2D-PCA plots), the first two principal component (PC) functions explained 51.4 and 25.2% of data variability, respectively. Figure 3 also shows that the data patterns only allowed a satisfactory by-product matrix differentiation (Figure 3(a)), being not successful for identifying the pomegranate cultivar (Figure 3(b)) neither the ethanol/water extraction solvent used (Figure 3(c)). On the other hand, Figure 3(d) strengthens that when the by-product and cultivar are simultaneously considered, the extracts obtained from the Big Full by-products can be easily split from those of the other two cultivars, which showed a higher similarity in terms of the parameters evaluated. Extracts of Big Full peels are the richest in TF and TPC and, oppositely, extracts obtained from seeds of Big Full cultivar are the poorest. These findings allowed inferring that the by-product matrix highly conditioned the extraction yield and the bioactive potential of the extracts, and the other two effects, although playing a key role, do not influence the studied parameters in a deep an undoubted manner that they could be used as cultivar or extraction solvent markers evaluated. According to the variables loadings (arrows) from Figure 3(a), it can be inferred that the highest EY, TPC, and TF are related with the peels' extracts, confirming the results of the three-way ANOVA. Figure 3(a) also pointed out that the peels' extracts had a higher dispersion in the 2D principal component space, compared to the seeds' extracts. This may hypothetically indicate that peel extraction is more prone to be differently influenced by cultivar and extraction conditions than seeds, allowing obtaining, with the latter, a more standardized extract composition, which may be advantageous if a future industrial application is envisaged, regardless the better bioactive overall potential of peels' extracts.

## 4. Conclusions

The study allowed concluding that the extraction yield (EY), total phenolic compounds (TPC), total flavonoids (TF) ,and antioxidant activity (AA, expressed as IC_50_) significantly depended on the type of pomegranate by-product (peels or seeds), on the pomegranate cultivar (Acco, Big Full, or Wonderful) and the relative proportion of ethanol/water used as solvent for extraction. Highest EY was achieved for pomegranate peels being the peels' extracts significantly richer in TPC and TF than seeds' extracts. Although seeds had lower IC_50_ in average (i.e., higher AA), Big Full cultivar had significantly greater overall TPC, TF, and AA demonstrating the potential production interest on this recent and less-studied pomegranate cultivar. EtOH 50% was the extraction solvent that promoted, as a whole, the highest average EY (45%), TPC (0.34 mg GAE/mg), and TF (0.026 mg CATE/mg), as well as the greatest AA (lowest IC_50_ 0.12 mg/mL). Finally, it was verified that, the experimental data collected (EY, TPC, TF, and IC_50_), enabled the unsupervised differentiation (principal component analysis) of pomegranate by-products (peels versus seeds).

## Figures and Tables

**Table 1:** Characterization of Acco, Big Full, and Wonderful peels and seeds.

	Peels			Seeds		
	Acco	Big Full	Wonderful	Acco	Big Full	Wonderful
Moisture (%)	17.8 ± 0.3	10.1 ± 0.1	19.1 ± 0.2	21.6 ± 0.0	14.1 ± 0.1	15.7 ± 0.4
Ash (%, db)	2.89 ± 0.25	3.67 ± 0.11	3.13 ± 0.01	3.13 ± 0.15	4.39 ± 0.00	2.56 ± 0.04

Results are expressed as mean values ± standard deviation of duplicates of two assays.

**Table 2:** Qualitative phytochemical screening of pomegranate peel and seed extracts according to the solvent used for extraction and the pomegranate cultivar.

Solvent	By-product	Cultivar	TPC	TF	Tan	Sap	FA	CA	Alk	Pol	Terp	CG
EtOH 0%	Peels	Acco	+ + +	+ + +	+ + +	+	—	—	—	—	+	—
		Big Full	+ + +	+ + +	+ + +	+	—	+	—	—	+ +	—
		Wonderful	+ + +	+ + +	+ + +	+	—	—	—	—	+	—
	Seeds	Acco	+ + +	+ +	+ +	—	—	+ +	—	—	—	—
		Big Full	+ + +	+ +	—	—	+	+ + +	—	—	—	—
		Wonderful	+ + +	+ +	+ + +	—	—	+	—	—	+	—
EtOH 25%	Peels	Acco	+ + +	+ + +	+ + +	+ +	—	—	—	—	+	—
		Big Full	+ + +	+ + +	+ + +	+	—	+	—	—	+ + +	—
		Wonderful	+ + +	+ + +	+ + +	+ +	—	—	—	—	+ +	—
	Seeds	Acco	+ + +	+ +	+ +	—	—	+	—	—	+	—
		Big Full	+ + +	+ +	+	—	+ +	+ + +	—	—	—	—
		Wonderful	+ + +	+ +	+ + +	—	—	—	—	—	+	—
EtOH 50%	Peels	Acco	+ + +	+ + +	+ + +	+ +	—	—	—	—	—	—
		Big Full	+ + +	+ + +	+ + +	+ +	—	—	—	—	+	+
		Wonderful	+ + +	+ + +	+ + +	+ +	—	—	—	—	+	+
	Seeds	Acco	+ + +	+ +	+ + +	+	—	+	—	—	—	—
		Big Full	+ + +	+ +	+ + +	—	+ + +	+ + +	—	—	+	+ +
		Wonderful	+ + +	+ + +	+ + +	—	—	—	—	—	+	+
EtOH 75%	Peels	Acco	+ + +	+ + +	+ + +	—	—	—	—	—	+	+
		Big Full	+ + +	+ + +	+ + +	—	—	+	—	—	+ +	+ +
		Wonderful	+ + +	+ + +	+ + +	—	—	—	—	—	+	+ +
	Seeds	Acco	+ + +	+ +	+ + +	—	—	+	—	—	—	—
		Big Full	+ + +	+ +	+ +	—	+ + +	+ + +	—	—	—	—
		Wonderful	+ + +	+ + +	+ + +	—	—	—	—	—	+ +	+ +

+++: large response; ++: moderate response; +: minor response; -: no response. Results are expressed as mean values of duplicates of two independent extractions. TPC: total phenolic compounds; TF: total flavonoids; Tan: tannins; Sap: saponins; FA: free anthraquinones; CA: combined anthraquinones; Alk: alkaloids; Pol: polysteroids; Terp: triterpenoids; CG: cardiac glycosides.

**Table 3:** Extraction yield (EY, %), total phenolic compounds (TPC, mg GAE/mg extract), total flavonoids (TF, mg CATE/mg extract), and antioxidant activity (AA expressed as IC_50_, mg/mL) of the peels and seeds of three pomegranate cultivars (Acco, Big Full, Wonderful) according to ethanol/water extraction solvent (0%, 25%, 50%, and 75% EtOH, v/v).

Parameter	Solvent	Peels			Seeds		
		Acco	Big Full	Wonderful	Acco	Big Full	Wonderful
EY	EtOH 0%	43.4 ± 1.5	42.4 ± 6.9	44.8 ± 5.7	50.1 ± 1.4	34.4 ± 2.9	25.3 ± 3.4
	EtOH 25%	43.2 ± 3.1	47.0 ± 2.3	44.4 ± 4.0	54.0 ± 0.9	31.1 ± 2.4	27.5 ± 1.5
	EtOH 50%	49.9 ± 0.9	51.0 ± 0.3	46.3 ± 4.8	59.2 ± 1.3	35.7 ± 1.2	32.0 ± 1.7
	EtOH 75%	53.2 ± 2.7	54.8 ± 2.2	57.9 ± 5.4	53.0 ± 1.8	31.6 ± 0.4	30.2 ± 1.5
TPC	EtOH 0%	0.36 ± 0.02	0.39 ± 0.02	0.24 ± 0.02	0.09 ± 0.00	0.08 ± 0.01	0.13 ± 0.03
	EtOH 25%	0.36 ± 0.00	0.56 ± 0.15	0.25 ± 0.01	0.14 ± 0.01	0.12 ± 0.02	0.20 ± 0.01
	EtOH 50%	0.39 ± 0.02	0.73 ± 0.18	0.32 ± 0.01	0.21 ± 0.03	0.16 ± 0.03	0.23 ± 0.00
	EtOH 75%	0.40 ± 0.01	0.86 ± 0.12	0.28 ± 0.03	0.12 ± 0.01	0.10 ± 0.00	0.20 ± 0.00
TF	EtOH 0%	0.031 ± 0.003	0.047 ± 0.004	0.030 ± 0.001	0.007 ± 0.002	0.007 ± 0.002	0.021 ± 0.002
	EtOH 25%	0.036 ± 0.003	0.055 ± 0.002	0.030 ± 0.002	0.006 ± 0.002	0.010 ± 0.001	0.026 ± 0.001
	EtOH 50%	0.029 ± 0.002	0.052 ± 0.003	0.042 ± 0.005	0.008 ± 0.000	0.007 ± 0.001	0.021 ± 0.001
	EtOH 75%	0.027 ± 0.001	0.059 ± 0.003	0.030 ± 0.004	0.004 ± 0.002	0.007 ± 0.001	0.014 ± 0.002
AA	EtOH 0%	6.606 ± 0.032	1.095 ± 0.018	5.154 ± 0.034	1.119 ± 0.363	1.133 ± 0.376	0.286 ± 0.004
	EtOH 25%	0.031 ± 0.001	0.142 ± 0.023	0.022 ± 0.002	0.063 ± 0.002	0.394 ± 0.057	0.045 ± 0.001
	EtOH 50%	0.024 ± 0.000	0.180 ± 0.005	0.022 ± 0.001	0.063 ± 0.004	0.398 ± 0.017	0.042 ± 0.003
	EtOH 75%	0.023 ± 0.003	0.196 ± 0.005	0.021 ± 0.001	0.045 ± 0.005	0.556 ± 0.033	0.038 ± 0.005

Results are expressed as mean values ± standard deviation of four independent extractions for EY and duplicates of two independent extractions for TPC, TF and AA.

**Table 4:** Three-way ANOVA: influence of pomegranate by-product (peels and seeds), pomegranate cultivar (Acco, Big Full, Wonderful), and ethanol/water extraction solvent (0%, 25%, 50%, and 75% EtOH, v/v) on the extraction yield (EY), total phenolic compounds (TPC), total flavonoids (TF), and antioxidant activity (AA expressed as IC_50_).

Factor	Levels	EY (%)^#^	TPC (mg GAE/mg extract)^#^	TF (mg CATE/mg extract)^#^	IC_50_ (mg/mL)^#^
Pomegranate by-product (A)	Peels	48 ± 6a	0.41 ± 0.18a	0.039 ± 0.011a	1.13 ± 2.21A
	Seeds	38 ± 11b	0.15 ± 0.05b	0.012 ± 0.007b	0.35 ± 0.41B
	*P* value	<0.0001	<0.0001	<0.0001	<0.0001
Pomegranate cultivar (B)	Acco	51 ± 6a	0.26 ± 0.13b	0.018 ± 0.013c	1.00 ± 2.22A
	Big Full	41 ± 9b	0.36 ± 0.29a	0.031 ± 0.023a	0.51 ± 0.40C
	Wonderful	38 ± 11c	0.23 ± 0.06b	0.026 ± 0.008b	0.70 ± 1.74B
	*P* value	<0.0001	<0.0001	<0.0001	<0.0001
Extraction solvent (C)	EtOH 0%	40 ± 9b	0.21 ± 0.13c	0.024 ± 0.014b	2.57 ± 2.51A
	EtOH 25%	41 ± 10b	0.29 ± 0.18b	0.027 ± 0.018a	0.12 ± 0.14B
	EtOH 50%	45 ± 10a	0.34 ± 0.20a	0.026 ± 0.017a	0.12 ± 0.14B
	EtOH 75%	46 ± 12a	0.30 ± 0.23a,b	0.024 ± 0.019b	0.15 ± 0.20B
	*P* value	<0.0001	<0.0001	<0.0001	<0.0001
A × B interaction	*P* value	<0.0001	<0.0001	<0.0001	<0.0001
A × C interaction	*P* value	<0.0001	0.0111	<0.0001	<0.0001
B × C interaction	*P* value	0.1205	0.0016	<0.0001	<0.0001
A × B × C interaction	*P* value	0.0806	<0.0001	<0.0001	<0.0001

Results are expressed as mean values ± standard deviation of four independent extractions for EY and duplicates of two independent extractions for TPC, TF, and IC_50_. ^#^If a nonsignificant or a significant additive 2^nd^ order interaction effect was found (EY, TPC, and TF), different lowercase letters in the same column indicate significant statistical differences between pomegranate by-products, pomegranate cultivars, or extraction solvent, according to Tukey's multiple range test (*P* value <0.05). If a disordinal 2^nd^ order interaction effects were observed (IC_50_), Tukey's multiple range test was conditionally performed (different uppercase letters in the same column indicate possible significant statistical differences), the discussion took into account, and the experimental trends observed on the estimated margin plots.

## Data Availability

The datasets generated during and/or analyzed during the current study that are not included in this article (neither in the supplementary information files) are available from the corresponding author upon reasonable request.

## Supplementary Materials

Supplementary Materials Figure S1: estimated marginal mean plots for the 2^nd^ order interactions of a three-way ANOVA, regarding the IC_50_ data determined for extracts of pomegranate by-products (peels and seeds) of three cultivars (Acco, Big Full, and Wonderful) obtained using different ethanol/water solvents (EtOH: 0, 25, 50, and 75%). Results are expressed as mean values of four independent extractions for EY and duplicates of two independent extractions for TPC, TF, and AA.

## References

[1] Stover E., Mercure E. W. (2007). The pomegranate: a new look at the fruit of paradise. *HortScience*.

[2] Panth N., Manandhar B., Paudel K. R. (2017). Anticancer activity of *Punica granatum* (pomegranate): a review. *hytotherapy research*.

[3] Passafiume R., Perrone A., Sortino G. (2019). Chemical-physical characteristics, polyphenolic content and total antioxidant activity of three Italian-grown pomegranate cultivars. *NFS journal*.

[4] Venkitasamy C., Zhao L., Zhang R., Pan Z., Pan Z., Zhang R., Zicari S. (2019). *Pomegranate, in Integrated Processing Technologies for Food and Agricultural By-Products*.

[5] Bassiri-Jahromi S. (2018). *Punica granatum* (pomegranate) activity in health promotion and cancer prevention. *Oncology Reviews*.

[6] Loizzo M. R., Aiello F., Tenuta M. C., Leporini M., Falco T., Tundis R., Nabavi S. M., Silva A. S. (2019). Pomegranate (*Punica granatum* L.). *Nonvitamin and Nonmineral Nutritional Supplements, 2*.

[7] INE - Instituto Nacional de Estatística (2019). Estatísticas Agrícolas -2018.

[8] Faria A., Calhau C., Watson R. R., Preedy V. R. (2010). Pomegranate in human health: an overview. *Bioactive Foods in Promoting Health*.

[9] Elfalleh W., Hannachi H., Tlili N., Yahia Y., Nasri N., Ferchichi A. (2012). Total phenolic contents and antioxidant activities of pomegranate peel, seed, leaf and flower. *Journal of Medicinal Plants Research*.

[10] Alexandre E. M. C., Silva S., Santos S. A. O. (2019). Antimicrobial activity of pomegranate peel extracts performed by high pressure and enzymatic assisted extraction. *Food Research International*.

[11] Duman A. D., Ozgen M., Dayisoylu K. S., Erbil N., Durgac C. (2009). Antimicrobial activity of six pomegranate (*Punica granatum* L.) varieties and their relation to some of their pomological and phytonutrient characteristics. *Molecules*.

[12] Goula A. M., Adamopoulos K. G. (2012). A method for pomegranate seed application in food industries: seed oil encapsulation. *Food and Bioproducts Processing*.

[13] Legua P., Forner-Giner M. Á., Nuncio-Jáuregui N., Hernández F. (2016). Polyphenolic compounds, anthocyanins and antioxidant activity of nineteen pomegranate fruits: a rich source of bioactive compounds. *Journal of Functional Foods*.

[14] Singh B., Singh J. P., Kaur A., Singh N. (2018). Phenolic compounds as beneficial phytochemicals in pomegranate (*Punica granatum* L.) peel: a review. *Food Chemistry*.

[15] Fourati M., Smaoui S., Ennouri K. (2019). Multiresponse optimization of pomegranate peel extraction by statistical versus artificial intelligence: predictive approach for foodborne bacterial pathogen inactivation. *Evidence-Based Complementary and Alternative Medicine*.

[16] Meselhy K. M., Shams M. M., Sherif N. H., El-sonbaty S. M. (2020). Phytochemical study, potential cytotoxic and antioxidant activities of selected food byproducts (pomegranate peel, rice bran, rice straw & mulberry bark). *Natural Product Research*.

[17] Mphahlele R. R., Fawole O. A., Makunga N. P., Linus Opara U. (2017). Functional properties of pomegranate fruit parts: influence of packaging systems and storage time. *Journal of Food Measurement and Characterization*.

[18] Masci A., Coccia A., Lendaro E., Mosca L., Paolicelli P., Cesa S. (2016). Evaluation of different extraction methods from pomegranate whole fruit or peels and the antioxidant and antiproliferative activity of the polyphenolic fraction. *Food Chemistry*.

[19] Karimi M., Sadeghi R., Kokini J. (2017). Pomegranate as a promising opportunity in medicine and nanotechnology. *Trends in Food Science and Technology*.

[20] Akhtar S., Ismail T., Fraternale D., Sestili P. (2015). Pomegranate peel and peel extracts: chemistry and food features. *Food Chemistry*.

[21] Derakhshan Z., Ferrante M., Tadi M. (2018). Antioxidant activity and total phenolic content of ethanolic extract of pomegranate peels, juice and seeds. *Food and Chemical Toxicology*.

[22] Fourati M., Smaoui S., Hlima H. B. (2020). Bioactive compounds and pharmacological potential of pomegranate (*Punica granatum*) seeds - a review. *Plant Foods for Human Nutrition*.

[23] Mushtaq M., Sultana B., Anwar F., Adnan A., Rizvi S. S. H. (2015). Enzyme-assisted supercritical fluid extraction of phenolic antioxidants from pomegranate peel. *Journal of Supercritical Fluids*.

[24] Hlima H. B., Bohli T., Kraiem M. (2019). Combined effect of *Spirulina platensis and Punica granatum* peel extacts: phytochemical content and antiphytophatogenic activity. *Applied Sciences*.

[25] Goula A. M., Ververi M., Adamopoulou A., Kaderides K. (2017). Green ultrasound-assisted extraction of carotenoids from pomegranate wastes using vegetable oils. *Ultrasonics Sonochemistry*.

[26] Canuti V., Cecchi L., Khatib M., Guerrini L., Mulinacci N., Zanoni B. (2020). A new extract from pomegranate (*Punica granatum* L.) by-products as a potential oenological tannin: preliminary characterization and comparison with existing commercial products. *Molecules*.

[27] Safari M., Ghasemi E., Alikhani M., Ansari-Mahyari S. (2018). Supplementation effects of pomegranate by-products on oxidative status, metabolic profile, and performance in transition dairy cows. *Journal of Dairy Science*.

[28] Campos L., Seixas L., Dias S., Peres A. M., Veloso A. C. A., Henriques M. (2022). Effect of extraction method on the bioactive composition, antimicrobial activity and phytotoxicity of pomegranate by-products. *Foods*.

[29] Cunniff P. (1997). *930.04. Moisture in Plants. Official Methods of Analysis of AOAC International*.

[30] Cunniff P. (1997). *930.05. Ash of Plants. Official Methods of Analysis of AOAC International*.

[31] Vesoul J., Cock I. (2011). An examination of the medicinal potential of Pittosporum phylliraeoides: toxicity, antibacterial and antifungal activities. *Pharmacognosy Communications*.

[32] Singleton V. L., Rossi J. (1965). Colorimetry of total phenolics with phosphomolybdic-phosphotungstic acid reagents. *American Journal of Enology and Viticulture*.

[33] Kim D., Jeong S. W., Lee C. Y. (2003). Antioxidant capacity of phenolic phytochemicals from various cultivars of plums. *Food Chemistry*.

[34] Kanatt S. R., Chander R., Sharma A. (2010). Antioxidant and antimicrobial activity of pomegranate peel extract improves the shelf life of chicken products. *International Journal of Food Science and Technology*.

[35] Smaoui S., Hlima H. B., Mtibaa A. C. (2019). Pomegranate peel as phenolic compounds source: advanced analytical strategies and practical use in meat products. *Meat Science*.

[36] Shaygannia E., Bahmani M., Zamanzad B., Rafieian-Kopaei M. (2016). A review study on *Punica granatum* L. *Journal of evidence-based complementary & alternative medicine*.

[37] Field A. (2009). *Discovering Statistics Using SPSS, 3rd Editio*.

[38] Cadima J., Cerdeira J. O., Minhoto M. (2004). Computational aspects of algorithms for variable selection in the context of principal components. *Computational Statistics and Data Analysis*.

[39] Venables W. N., Ripley B. D. (2002). *Modern applied statistics with S (statistics and computing), 4th Editio*.

[40] Güzel M., Akpınar Ö. (2019). Valorisation of fruit by-products: production characterization of pectins from fruit peels. *Food and Bioproducts Processing*.

[41] Ullah N., Ali J., Ali Khan F., Khurram M., Hussain A., Inayat-ur-Rahman; Zia-ur-Rahman; Shafqatullah (2012). Proximate composition, minerals content, antibacterial and antifungal activity evaluation of pomegranate (*Punica granatum L.*) peels powder, Middle East. *Journal of Scientific Research*.

[42] Jalal H., Pal M. A., Ahmad S. R., Rather M., Andrabi M., Hamdani S. (2018). Physico-chemical and functional properties of pomegranate peel and seed powder. *Journal of Pharmaceutical Innovation*.

[43] Rowayshed G., Salama A., Abul-Fadl M., Akila-Hamza S., Emad A. M. (2013). Nutritional and chemical evaluation for pomegranate (*Punica granatum* L.) fruit peel and seeds powders by products, Middle East. *Journal of Applied Sciences*.

[44] Wang Z., Pan Z., Ma H., Atungulu G. G. (2011). Extract of phenolics from pomegranate peels. *Open Food Science Journal*.

[45] Borges J. G., dos Santos Garcia V. A., de Carvalho R. A. (2019). Extraction of active compounds from different parts of pomegranate and incorporation into a potential delivery model system using a printing technique. *Food Bioscience*.

[46] Gözlekçi Ş., Saraçoǧlu O., Onursal E., Özgen M. (2011). Total phenolic distribution of juice, peel, and seed extracts of four pomegranate cultivars. *Pharmacognosy Magazine*.

[47] Orak H. H., Yagar H., Isbilir S. S. (2012). Comparison of antioxidant activities of juice, peel, and seed of pomegranate (*Punica granatum* L.) and inter-relationships with total phenolic, tannin, anthocyanin, and flavonoid contents. *Food Science and Biotechnology*.

[48] Falcinelli B., Marconi O., Maranghi S. (2017). Effect of genotype on the sprouting of pomegranate (*Punica granatum* L.) seeds as a source of phenolic compounds from juice industry by-products. *Plant Foods for Human Nutrition*.

[49] Malviya S., Jha A., Hettiarachchy N. (2014). Antioxidant and antibacterial potential of pomegranate peel extracts. *Journal of Food Science and Technology*.

[50] Rahnemoon P., Sarabi Jamab M., Javanmard Dakheli M., Bostan A., Safari O. (2018). Comparison of two methods of solvent extraction of phenolic compounds from pomegranate (*Punica granatum* L.) peels. *Journal of Agricultural Science and Technology*.

[51] Sabraoui T., Khider T., Nasser B. (2020). Determination of Punicalagins content, metal chelating, and antioxidant properties of edible pomegranate (*Punica granatum* L) peels and seeds grown in Morocco. *International Journal of Food Science*.

[52] Rummun N., Somanah J., Ramsaha S., Bahorun T., Neergheen-bhujun V. S. (2013). Bioactivity of nonedible parts of *Punica granatum* L.: a potential source of functional ingredients. *International Journal of Food Science*.

[53] Sumere B. R., de Souza M. C., dos Santos M. P. (2018). Combining pressurized liquids with ultrasound to improve the extraction of phenolic compounds from pomegranate peel (*Punica granatum* L.). *Ultrasonics Sonochemistry*.

[54] Santos M. P., Souza M. C., Sumere B. R. (2019). Extraction of bioactive compounds from pomegranate peel (*Punica granatum* L.) with pressurized liquids assisted by ultrasound combined with an expansion gas. *Ultrasonics Sonochemistry*.

[55] Qu W., Breksa A. P., Pan Z., Ma H., McHugh T. H. (2012). Storage stability of sterilized liquid extracts from pomegranate peel. *Journal of Food Science*.

[56] Venkataramanamma D., Aruna P., Singh R. P. (2016). Standardization of the conditions for extraction of polyphenols from pomegranate peel. *Journal of Food Science and Technology*.

[57] Sood A., Gupta M. (2015). Extraction process optimization for bioactive compounds in pomegranate peel. *Food Bioscience*.

[58] Al-Zoreky N. S. (2009). Antimicrobial activity of pomegranate (*Punica granatum* L.) fruit peels. *International Journal of Food Microbiology*.

[59] Hmid I., Elothmani D., Hanine H., Oukabli A., Mehinagic E. (2017). Comparative study of phenolic compounds and their antioxidant attributes of eighteen pomegranate (*Punica granatum* L.) cultivars grown in Morocco. *Arabian Journal of Chemistry*.

