# Supplementary material for: Pomegranate Peels and Seeds as a Source of Phenolic Compounds: Effect of Cultivar, By-Product, and Extraction Solvent
Source: Int J Food Sci. 2022 Jul 18;2022:9189575. doi: 10.1155/2022/9189575 (PMC9314001; doi:10.1155/2022/9189575)
Supplement: Supplementary Materials — Figure S1: estimated marginal mean plots for the 2nd order interactions of a three-way ANOVA, regarding the IC50 data determined for extracts of pomegranate by-products (peels and seeds) of three cultivars (Acco, Big Full, and Wonderful) obtained using different ethanol/water solvents (EtOH: 0, 25, 50, and 75%). Results are expressed as mean values of four independent extractions for EY and duplicates of two independent extractions for TPC, TF, and AA. [file 9189575.f1.docx]

**Supplementary Material**

**Pomegranate peels and seeds as a source of phenolic compounds: effect of cultivar, by-product and extraction solvent**

Lara Campos^1,2,3^, Luana Seixas^4^, Marta H.F. Henriques^1,2^, António M. Peres^5^, Ana C.A. Veloso^3,4,6,*^

^1^Polytechnic Institute of Coimbra, Coimbra Agriculture School, Bencanta, 3045-601 Coimbra, Portugal.

^2^CERNAS - Research Centre for Natural Resources, Environment and Society, Coimbra Agriculture School, Bencanta, 3045-601 Coimbra, Portugal.

^3^CEB - Centre of Biological Engineering, University of Minho, Campus de Gualtar, 4715-057 Braga, Portugal.

^4^Polytechnic Institute of Coimbra, Coimbra Institute of Engineering, Rua Pedro Nunes - Quinta da Nora, 3030-199 Coimbra, Portugal.

^5^Centro de Investigação de Montanha (CIMO), ESA, Instituto Politécnico de Bragança, Campus de Santa Apolónia, 5300-253 Bragança, Portugal.

^6^LABBELS - Associate Laboratory, Braga/Guimarães, Portugal

*Corresponding author:

Ana C.A. Veloso; E-mail: anaveloso@isec.pt; ORCID ID 0000-0001-8196-7624

Authors email's:

Lara Campos; E-mail: [lara.campos@esac.pt](mailto:lara.campos@esac.pt); ORCID ID 0000-0002-3430-1704

Luana Seixas: E-mail: [luana.seixas00@gmail.com](mailto:luana.seixas00@gmail.com); ORCID ID 0000-0003-1794-0149

Marta Henriques: E-mail: [mhenriques@esac.pt](mailto:mhenriques@esac.pt); ORCID ID 0000-0001-9214-0614

António Peres: E-mail address: [peres@ipb.pt](mailto:peres@ipb.pt); ORCID ID 0000-0001-6595-9165


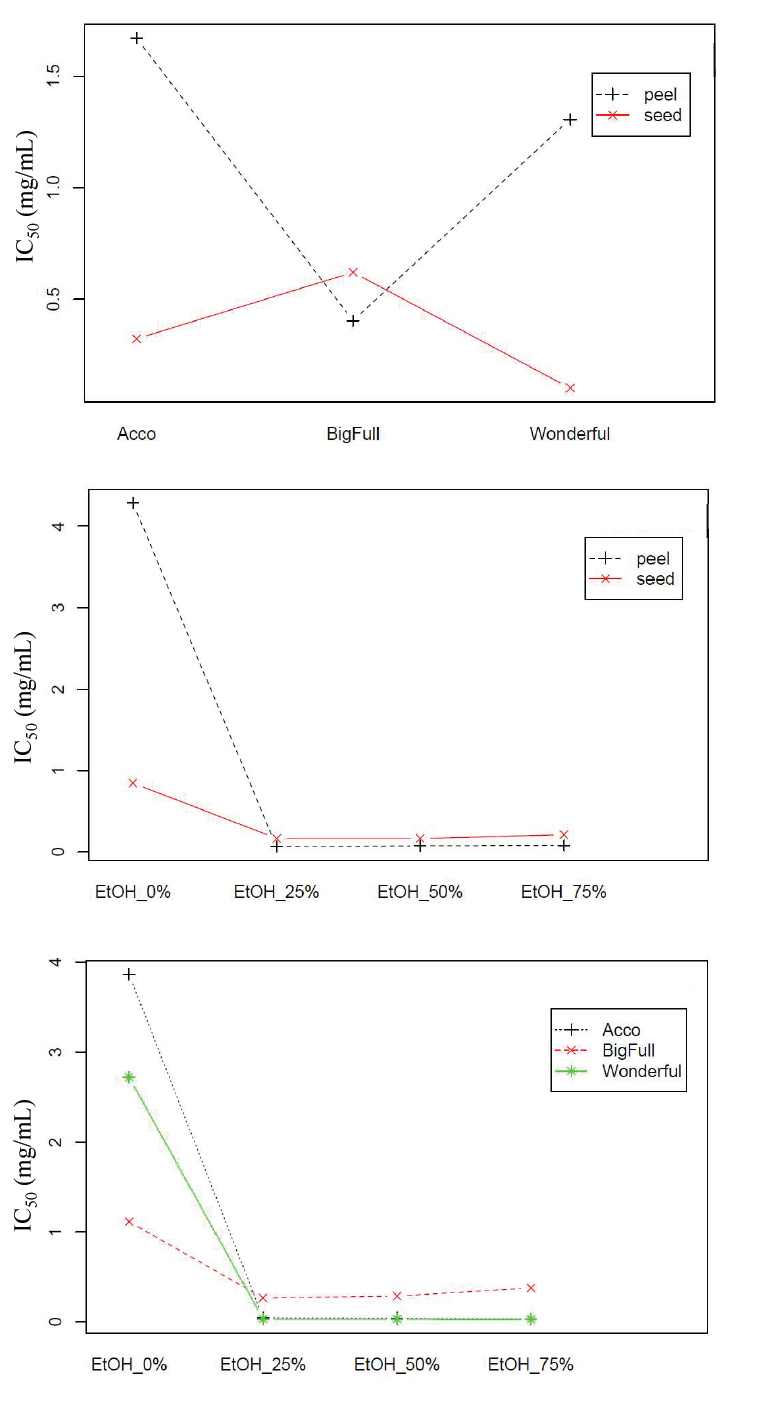
**Figure S1.** Estimated marginal mean plots for the 2^nd^ order interactions of a three-way ANOVA, regarding the IC_50_ data determined for extracts of pomegranate by-products (peels and seeds) of three cultivars (Acco, Big Full and Wonderful) obtained using different ethanol:water solvents (EtOH: 0, 25, 50 and 75%). Results are expressed as mean values of four independent extractions for EY and duplicates of two independent extractions for TPC, TF and AA.
